# Spontaneous Simultaneous Bilateral Basal Ganglia, Thalamic, and Central Pontine Haemorrhage: A Case Report

**DOI:** 10.7759/cureus.53674

**Published:** 2024-02-05

**Authors:** Husam Jamil, Jouher Kallingal

**Affiliations:** 1 Stroke Medicine, Stroke Unit, Salford Royal Hospital, Salford, GBR

**Keywords:** hyperacute stroke unit, hypertension, bilateral basal ganglia hyperintensities, pontine hemorrhage, hemorragic stroke

## Abstract

The basal ganglia, a complex of subcortical nuclei, form an important functional component of the brain. Spontaneous simultaneous bilateral basal ganglia haemorrhage (SSBBGH) is exceedingly uncommon and often associated with hypertension as a primary predisposing factor. We report a case of a 72-year-old female who presented to a local hospital following a dizzy spell and subsequent fall. Non-contrast CT brain revealed bilateral basal ganglia haemorrhage alongside central pontine haemorrhage. Subsequently, she was transferred to our tertiary-care specialist stroke unit where conservative management was pursued. She was discharged after brain imaging, multidisciplinary team (MDT) consultations, and follow-up plans. The MDT comprised stroke physicians, radiologists, physiotherapists, occupational therapists, and speech and language therapists. Given the limited number of documented cases of this rare occurrence (approximately 60 in the literature), we believe this report will contribute to the existing body of knowledge.

## Introduction

The basal ganglia are a subcortical group of structures in the brain involved in supporting voluntary movements, language, memory, decision-making, and procedural learning [1]. Anatomically, they include the striatum (which includes caudate and putamen nucleus), subthalamic nucleus, globus pallidus, and substantia nigra [1]. A haemorrhage in this region can lead to varied clinical presentations. Bilateral basal ganglia bleeding is rare [2], and as per a systematic review of 60 non-traumatic simultaneous bilateral basal ganglia haemorrhage (SBBGH) case reports [3], it is most commonly reported in Asia (68.3%) compared to other continents (31.6%). In this report, we discuss a case of spontaneous SBBGH (SSBBGH), bilateral thalamic haemorrhage, and pontine haemorrhage, which was managed conservatively. Our primary objective in reporting this case is to enhance the understanding of this condition by providing detailed insights into its clinical presentation, aetiology, diagnostic considerations, and management approaches. By sharing our findings and experience, we aim to highlight the importance of conservative management and a multidisciplinary team (MDT) approach for these patients.

## Case presentation

**Figure 1:**
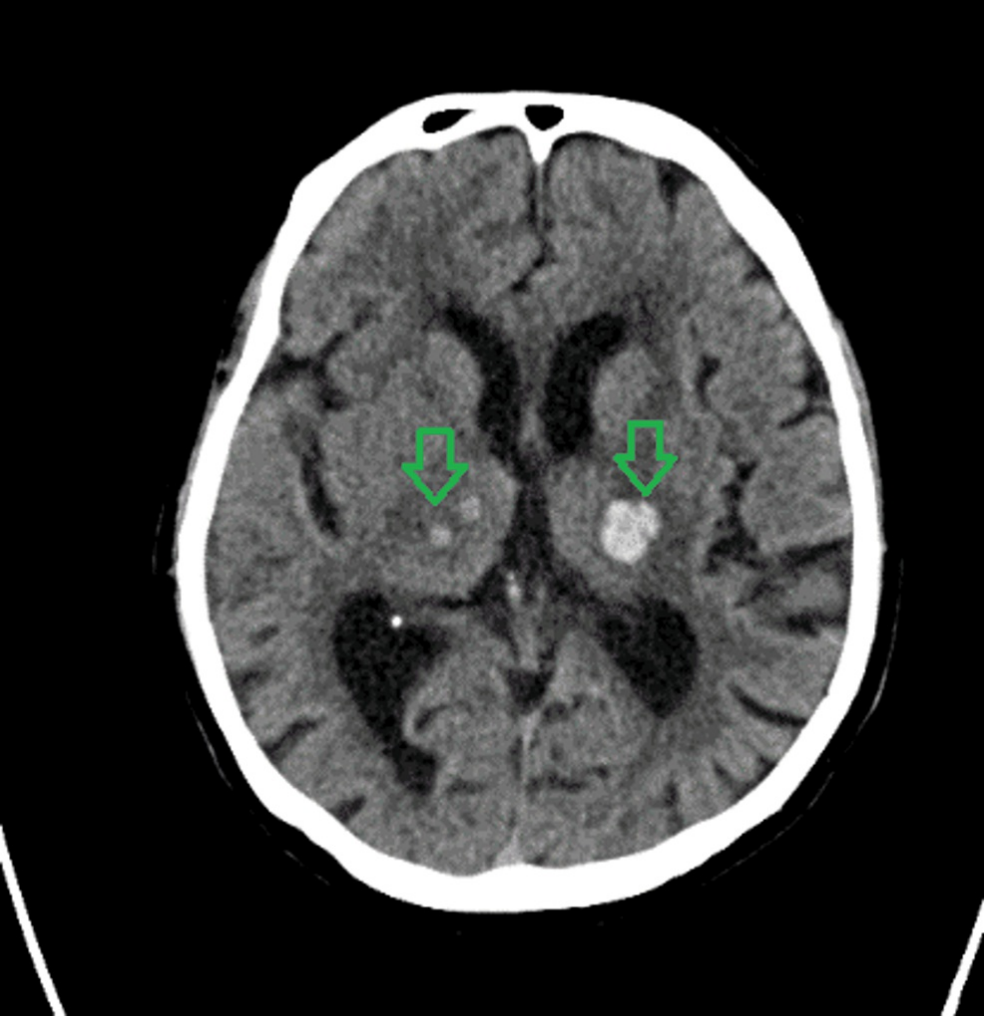
Acute hyperdensities in bilateral lateral thalamus regions with Hounsfield units of 58.16 (left) and 52 (right) suggestive of haemorrhage

**Figure 2:**
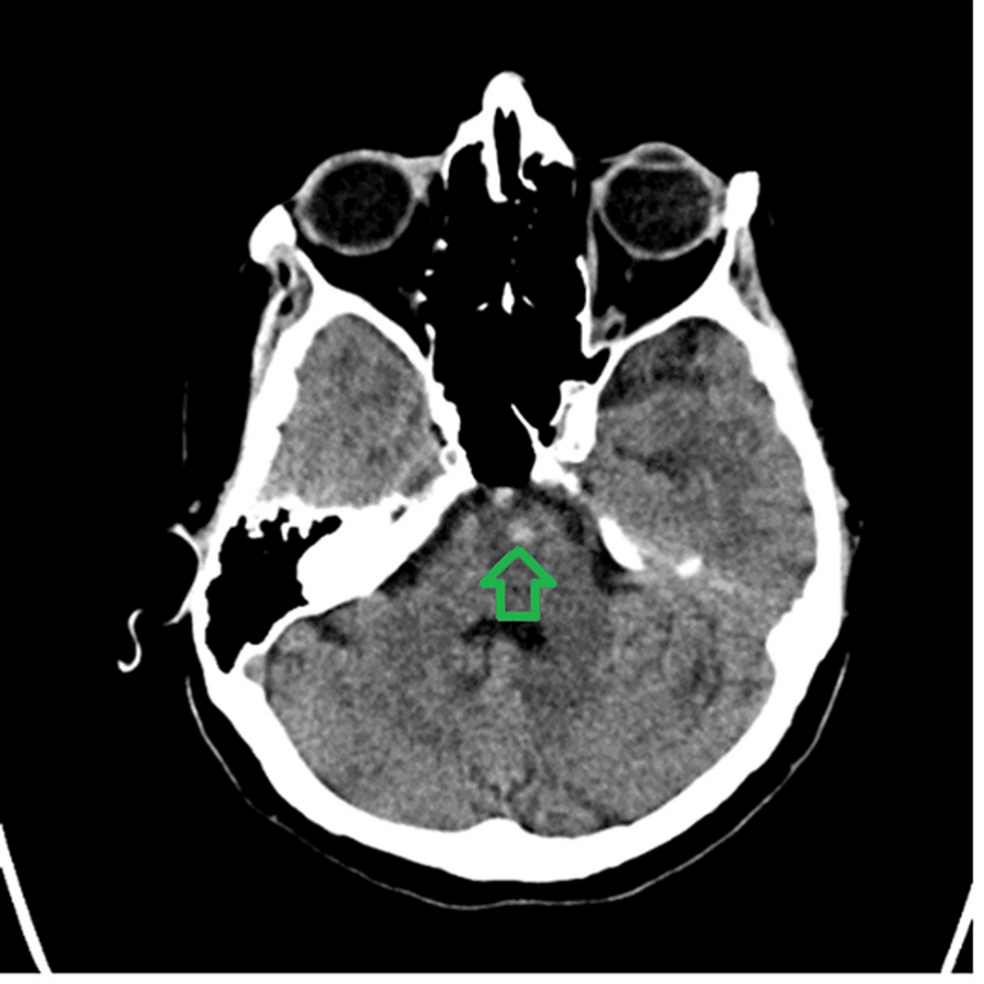
CT brain showing hyperdensity in anterior pons with 55 Hounsfield units suggestive of haemorrhage CT: computed tomography

A 72-year-old female of Asian ethnicity who lived in a sheltered accommodation presented to the local hospital in December 2022 following a fall after she had felt dizzy. There was no report of any head injury. A non-contrast CT head (Figures 1-2) was performed, which showed multiple focal haemorrhages in bilateral basal ganglia, thalamus capsular region, and central pons. She was then transferred to a regional stroke centre for further care.

She had a previous medical history of schizophrenia, diabetes mellitus, hypothyroidism, iron deficiency anaemia and Gilbert’s syndrome. She was not on any oral anticoagulant medications or any antiplatelet treatment. On examination, she had a pronator drift in both upper limbs. Power in the right lower limb as per the MRC scale was 4/5, while it was 5/5 in all other limbs. She also had an expressive dysphasia. The rest of the neurological exam was normal. Her National Institute of Health Stroke Scale (NIHSS) score was 4/42 and her Glasgow Coma Scale (GCS) was 14 (eyes: 4, motor: 6, and verbal: 4). ECG showed normal sinus rhythm. Her blood pressure was 172/80 mmHg on presentation.

**Figure 3:**
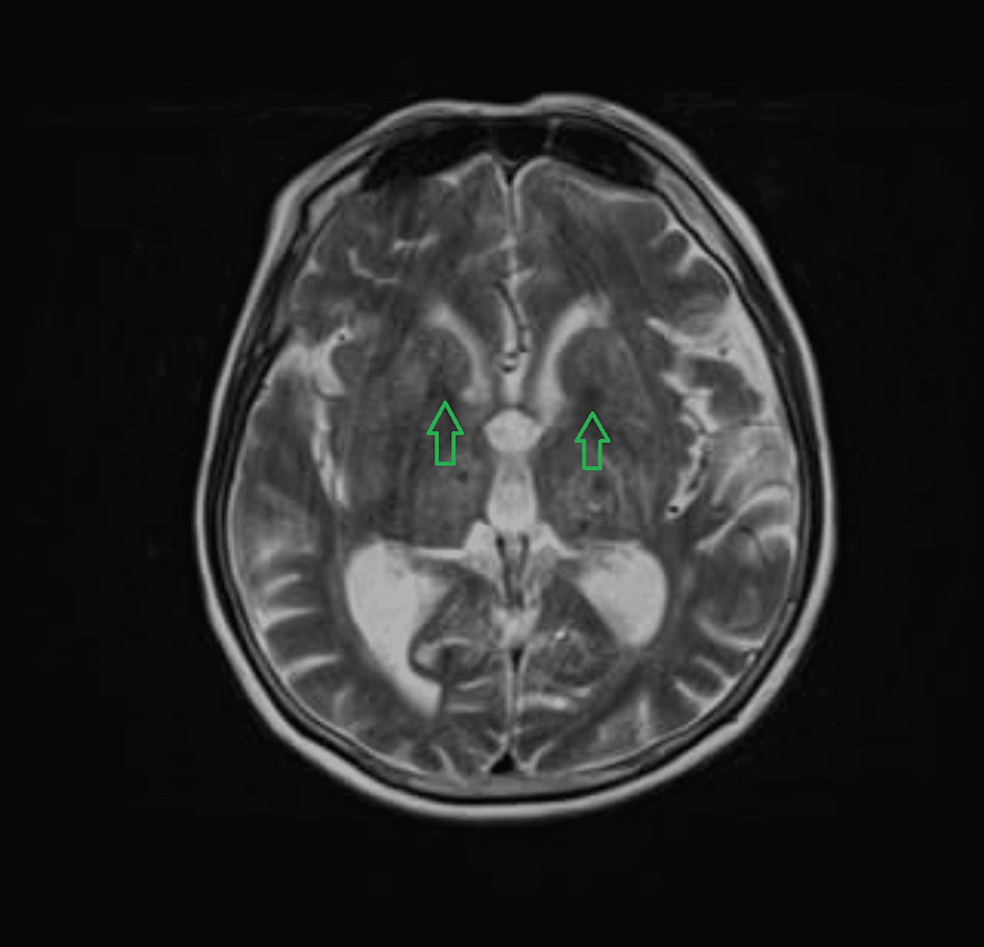
MRI brain indicating recent small haemorrhages in the bilateral basal ganglia region MRI: magnetic resonance imaging

**Figure 4:**
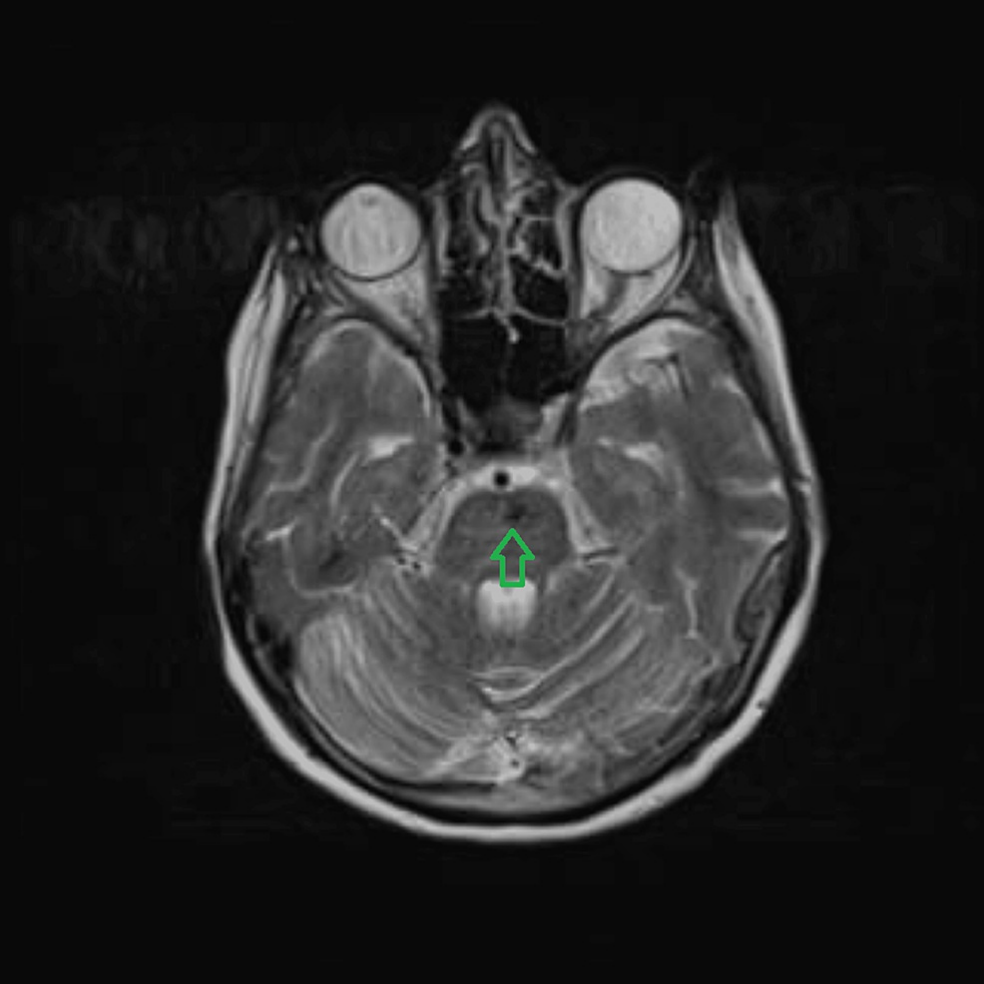
MRI brain showing pontine haemorrhage corresponding to CT head pontine haemorrhage CT: computed tomography; MRI: magnetic resonance imaging

She was admitted to hyper acute stroke unit (HASU) for further management. An MRI of the brain (Figures 3-4) was performed, uncovering regions of recent haemorrhage aligning with the observations on the CT scan, except for one small focus on the right side of the corpus callosum. This suggested that these haemorrhages may not be indicative of previous microbleeds, which are often associated with underlying conditions like cerebral amyloid angiopathy or chronic hypertensive encephalopathy. Instead, the current manifestation could be attributed to an acute episode of hypertension, although the exact cause remained uncertain.

Her elevated blood pressure resolved on its own and reached acceptable limits without any intervention. After a period of observation and MDT input, she was transferred to a local hospital for further rehabilitation and follow-up. Upon discharge, her modified Rankin scale (mRS) score stood at 4 out of 6. Subsequent follow-up one year post-discharge showed a sustained mRS score of 4 out of 6 and she was now residing at a nursing home due to increased care requirements. She remained alive with no scheduled further follow-up appointments thereafter.

## Discussion

Intracranial haemorrhages (ICH) account for 8-14% of initial stroke presentations and multiple ICHs have been reported in only 2% of haemorrhagic strokes [4]. SSBGH represents an uncommon form of ICH characterized by diverse presentations and is typically associated with variable and frequently unfavourable outcomes [2]. it is more common in males than females, with a male-to-female ratio of 2.3:1. Based on a systematic review [3] involving 60 documented cases, SSBGH has a higher prevalence among Asians (68.3%), particularly in Japanese and Indian populations, compared to people in other continents (31.6%). Our patient was also of Indian ethnicity.

Hypertension [5] is the primary contributor, accounting for 50% of cases in individuals aged 50 and older. The pathophysiology of long-standing hypertension is hypertensive vasculopathy, involving the formation of microaneurysms, known as Charcot-Bouchard aneurysms [3], in the lateral lenticulostriate arteries. Hypertension was also considered the primary cause of presentation in our patient and the rationale for this is discussed further below in this article.

Conversely, in younger patients aged below 50 years who present with normal blood pressure, various alternative etiopathogenic factors [3], such as metabolic and intoxication, infectious, and vascular causes, were found to be more prevalent. In the metabolic and intoxication group [3], which demonstrated an 18.33% prevalence, alcohol emerged as the primary intoxicating substance, involved in 72.7% of instances. Other contributing factors [3] included intoxication from olanzapine, diabetic ketoacidosis, and hyperglycaemia hyperosmolar syndrome, each appearing in a single case. Underlying vascular causes [3] were identified in only 16.66% of instances, with infection-related SSBGH being the least frequent, with merely 10% of reported cases. The infections included [3] coronavirus disease 2019 (COVID-19)-related encephalitis, toxoplasmosis encephalitis, dengue encephalitis, mucormycosis-caused encephalitis, and Japanese encephalitis. Interestingly, a definitive cause remained elusive in just two patients [3], highlighting the potential for the coexistence of multiple etiological factors.

The differential diagnoses for this presentation include cerebral venous sinus thrombosis, cerebral amyloid angiopathy [4], arteriovenous malformations, brain tumour, or metastasis and specific infections such as COVID-19 encephalitis and toxoplasmosis encephalitis. The weekly neuroradiology MDT meeting, which included neuroradiologists and stroke physicians, thoroughly reviewed the scans and the above differentials were considered. A non-contrast CT scan of the head revealed multiple focal haemorrhages in the bilateral basal ganglia, thalamus capsular region, and central pons and a subsequent MRI confirmed the areas of recent haemorrhage consistent with the CT findings, except for a small focus in the right side of the corpus callosum. This suggested that the haemorrhages were unlikely to be indicative of previous microbleeds associated with conditions such as cerebral amyloid angiopathy or chronic hypertensive encephalopathy. Upon reviewing the CT and MRI results, the consensus among the panel was that hypertension was the most probable cause of the patient's presentation. Consequently, no further vascular imaging was deemed necessary.

Traumatic basal ganglia haemorrhage accounts for a mere 3% of individuals with closed head injuries, and the occurrence of traumatic bilateral basal ganglia haemorrhage is even less frequent [6]. Recognized causes of traumatic bilateral basal ganglia haemorrhage include neurosurgical procedures and incidents like lightning strikes [7]. Importantly, our patient had not experienced any head injury before the onset of symptoms. SSBBGH can have varied clinical manifestations. The most common reported features [3] of SSBBGH are headache, focal neurological deficit, and reduced level of consciousness, observed in 78.33%, 46.66% and 25% of cases, respectively. Seizures are rare and the underlying disease process leading to SSBGH could manifest [3] as abdominal pain, vomiting, and fever.

Our patient’s main presentation involved dizziness, expressive dysphasia, mild confusion, pronator drift in both upper limbs and 4/5 MRC scale power in the right lower limb. Her symptoms improved with ongoing conservative management. Haemorrhage in the bilateral basal ganglia can cause [1] motor dysfunction, resulting in weakness and altered muscle tone, impacting both sides of the body. In the bilateral lateral thalamus, the involvement disrupts sensory and motor signal relay, potentially causing sensory abnormalities and coordination issues, manifesting as dizziness and mild confusion. Also, damage to this area may interfere with the intricate processes of language formation and expression [1]. The anterior pons, essential for motor control and coordination, may be affected by haemorrhage, influencing specific pathways that control the upper and lower limbs. This can explain the pronator drift and diminished power in the right lower limb [1]. Hence the symptoms of our patient could be explained based on sites of haemorrhages on various neuroanatomical structures on brain imaging.

The CT scan showed pontine haemorrhage alongside SSBBGH. Our review of the literature did not reveal any case of SBBGH with pontine haemorrhage and perhaps this combination of ICH in our patient is the first case of its kind to be reported. Typically, a pontine haemorrhage may present as respiratory failure, pinpoint pupils, tetraplegia, and decerebrate rigidity [8], but our patient had none of these features. The patient's pertinent medical history regarding stroke included a diagnosis of type 2 diabetes. Research aiming to establish a connection between type 2 diabetes and intracerebral haemorrhage has yielded varied results. Some studies [9] have indicated a significant correlation between diabetes and ICH, identifying diabetes as a notable risk factor for this condition. Conversely, other studies [10] have suggested that there is no link between type 2 diabetes mellitus and ICH. Our patient's blood glucose and HbA1c levels were within the normal range, attributed to diligent medication adherence. Therefore, we did not consider diabetes as a contributing factor to the current presentation.

The mortality rate for spontaneous SSBBGH is 33.33% as per the systematic review by Alhashim et al. [3], which is comparable to the mortality rates observed in patients with primary unilateral basal ganglia haemorrhage, falling within the range of 37-46%. This high mortality can be a result of the size and extension of the haemorrhage. Conservative management [2,3,4] has been the mainstay of treatment in the reported cases. The management strategies [3] include blood pressure-lowering regimens, coagulopathy reversal, surgical input for complication management, and using an MDT approach.

Following a period of monitoring and therapeutic intervention by physiotherapists, occupational therapists and speech and language therapists, our patient demonstrated clinical improvement and was transferred back to a local hospital for ongoing care. At the time of discharge, her mRS score was 4 out of 6. Subsequent follow-up one-year post-discharge revealed a consistent mRS score of 4 out of 6, and she remained alive. She is currently residing in a nursing home with care needs related to washing, dressing, and toilet use. This aligns with a systematic review's [3] finding that, among the survivors, only 40.6% (13 out of 32 cases) experienced positive outcomes (mRS score ≤2), while the remaining 59.4% (19 out of 32) exhibited unfavourable functional statuses (mRS score ≥3-5).

Our treatment strategy of conservative management was in line with that described in other case reports [2] and the systematic review by Alhashim et al. [3] as the best management approach for the patient. Further studies are needed to gain deeper insights into and enhance our understanding of this rare presentation.

## Conclusions

SSBBGH is an unusual condition with distinctive clinical and radiological features and is more common in Asian populations. Its aetiology may involve deep cerebral venous sinus thrombosis, cerebral amyloid angiopathy in the setting of cortical involvement, hypertension, alcohol intake, and certain infections. Our patient's case highlights the variability in its clinical presentation, as she exhibited expressive dysphasia, bilateral pronator drift, dizziness, and mild confusion-related symptoms that can be explained by haemorrhage in the bilateral basal ganglia, anterior pons, and thalami. The absence of microbleeds on MRI, compared to CT, can serve as a clue to differentiate between acute and old micro-haemorrhages. Conservative management, with a focus on blood pressure control, is crucial, especially when hypertension is the primary cause. However, in younger patients without a history of hypertension, a thorough investigation to identify the underlying cause is essential. The mortality rate associated with this condition is high, underscoring the importance of an MDT approach for accurate diagnosis and management.
